# Objectively Measured Physical Activity in European Adults: Cross-Sectional Findings from the Food4Me Study

**DOI:** 10.1371/journal.pone.0150902

**Published:** 2016-03-21

**Authors:** Cyril F. M. Marsaux, Carlos Celis-Morales, Jettie Hoonhout, Arjan Claassen, Annelies Goris, Hannah Forster, Rosalind Fallaize, Anna L. Macready, Santiago Navas-Carretero, Silvia Kolossa, Marianne C. Walsh, Christina-Paulina Lambrinou, Yannis Manios, Magdalena Godlewska, Iwona Traczyk, Julie A. Lovegrove, J. Alfredo Martinez, Hannelore Daniel, Mike Gibney, John C. Mathers, Wim H. M. Saris

**Affiliations:** 1 Department of Human Biology, NUTRIM School of Nutrition and Translational Research in Metabolism, Maastricht University Medical Centre + (MUMC+), Maastricht, The Netherlands; 2 Human Nutrition Research Centre, Institute of Cellular Medicine, Newcastle Upon Tyne, United Kingdom; 3 Experiences Research Department, Philips Research, Eindhoven, The Netherlands; 4 Philips Innovation Services, Software Department, Eindhoven, The Netherlands; 5 Personal Health Solutions, Philips Consumer Lifestyle, Amsterdam, The Netherlands; 6 UCD Institute of Food and Health, University College Dublin, Belfield, Dublin 4, Republic of Ireland; 7 Hugh Sinclair Unit of Human Nutrition and Institute for Cardiovascular and Metabolic Research, University of Reading, Reading, United Kingdom; 8 Department of Nutrition, Food Science and Physiology, Centre for Nutrition Research, University of Navarra, Pamplona, Spain; 9 CIBER Fisiopatogía de la Obesidad y Nutrición (CIBERobn), Instituto de Salud Carlos III, Madrid, Spain; 10 ZIEL Research Center of Nutrition and Food Sciences, Biochemistry Unit, Technische Universität München, München, Germany; 11 Department of Nutrition and Dietetics, Harokopio University, Athens, Greece; 12 National Food & Nutrition Institute (IZZ), Warsaw, Poland; University of South Australia, AUSTRALIA

## Abstract

**Background:**

Comparisons of objectively measured physical activity (PA) between residents of European countries measured concurrently with the same protocol are lacking. We aimed to compare PA between the seven European countries involved in the Food4Me Study, using accelerometer data collected remotely via the Internet.

**Methods:**

Of the 1607 participants recruited, 1287 (539 men and 748 women) provided at least 3 weekdays and 2 weekend days of valid accelerometer data (TracmorD) at baseline and were included in the present analyses.

**Results:**

Men were significantly more active than women (physical activity level = 1.74 vs. 1.70, *p* < 0.001). Time spent in light PA and moderate PA differed significantly between countries but only for women. Adherence to the World Health Organization recommendation to accumulate at least 150 min of moderate-equivalent PA weekly was similar between countries for men (range: 54–65%) but differed significantly between countries for women (range: 26–49%). Prevalence estimates decreased substantially for men and women in all seven countries when PA guidelines were defined as achieving 30 min of moderate and vigorous PA per day.

**Conclusions:**

We were able to obtain valid accelerometer data in real time via the Internet from 80% of participants. Although our estimates are higher compared with data from Sweden, Norway, Portugal and the US, there is room for improvement in PA for all countries involved in the Food4Me Study.

## Introduction

Physical inactivity has been estimated to be responsible for more than 5.3 million deaths worldwide [1]. Moreover, among European men and women, approximately 7.3% of all deaths in 2008 might be attributable to inactivity compared with 3.7% to obesity [2] and there is strong evidence to suggest that even small increases in physical activity (PA) would lower the risk for many non-communicable diseases [1–3]. Yet, levels of PA across populations remain low [4]. To tackle this public health issue, the US Centers for Disease Control and Prevention and the American College of Sports Medicine produced standardized PA guidelines 20 years ago [5]. Since then, the World Health Organization (WHO), the European Union, and most countries around the world, have included PA guidelines in their health policies. Guidelines for Americans and Europeans have been updated to include recommendations for adolescents and for older adults [6–9]. For adults aged 18–64 years old, the WHO recommends a minimum of 150 min of moderate intensity PA per week, 75 min of vigorous intensity PA or an equivalent amount of moderate and vigorous PA (MVPA) [9].

In 2008, 34.8% of adults 15 years or older were insufficiently active in Europe [4]. Regular surveillance is needed to update these prevalence estimates and to evaluate the effectiveness of PA policies and promotion programs in European countries. In this context, the objective assessment of PA is a key issue. Prevalence of physical inactivity has been mainly derived from self-reported measures such as the Baecke questionnaire [10] or the International Physical Activity Questionnaire (IPAQ) [11]. These questionnaires have been, and still are, widely used due to their simple administration and low cost [12]. However, PA is frequently misreported, which leads to considerable measurement error [13–15]. Accelerometers offer a potential solution because they measure PA objectively. Given that they are small and easy to wear, store data up to several weeks and are acceptable in terms of reliability, these devices are now used increasingly in large studies to assess PA in children, adolescents and adults [16]. Although some European countries have reported adherence to PA guidelines using accelerometers in large cohorts [17–19], comparisons between European countries measured according to the same standardized protocols and concurrently are lacking.

Between 2012 and 2014, PA was assessed objectively by accelerometry in the participants of the Food4Me Proof-of-Principle (PoP) study. The Food4Me Study was a web-based randomized controlled trial on personalized nutrition, across seven European countries: Germany, Greece, Ireland, The Netherlands, Poland, Spain and the United Kingdom. The aim of the current paper is to describe and compare PA in adults from these countries, and evaluate adherence to PA guidelines, using baseline data from the Food4Me PoP study.

## Methods

### Subjects

Subjects were participants in the Food4Me Proof-of-Principle (PoP) study (www.food4me.org), a multi-center randomized controlled trial on personalized nutrition (ClinicalTrials.gov, registration number: NCT01530139). A total of 1607 healthy adults (653 men and 954 women) from seven European countries (Germany, Greece, Ireland, The Netherlands, Poland, Spain, and the United Kingdom) were randomized to the study between August 2012 and August 2013. As outlined elsewhere [20], subjects were ineligible to take part in the study if they were <18 years of age, had no or limited access to the Internet, were following a prescribed diet, or had altered nutritional requirements because of medical conditions. Subjects were recruited locally and nationally via the Internet, radio, newspapers, posters, flyers, social media and word of mouth. The ethics committee from each recruiting center (Technische Universität München, Harokopio University, University College Dublin, Maastricht University, Instytut Żywności i Żywienia, University of Navarra and University of Reading) approved the study protocol. All subjects provided informed written consent digitally before participating in the study.

### Study design

The design of the Food4Me PoP study has been described elsewhere [20]. Briefly, the study was web-based and therefore participants did not visit the recruiting centers. Anthropometric measurements were completed at home and questionnaires completed online. Participants received study kits by post, containing all necessary materials (including an accelerometer) to perform measurements at home, but used their own scales to measure body weight. Printed instructions were included in the kits and participants were reminded that explanatory videos demonstrating each measurement were also available on the Food4Me website. On the morning of their baseline measurement day, fasted participants self-measured their height, weight and waist circumference, and uploaded their measurement values directly on their personal Food4Me web page. In addition, they started wearing an accelerometer. The baseline PA assessment period lasted 2–3 weeks at which time participants were instructed to upload their accelerometer data to the Food4Me website using their own computer (*See the ‘objective PA assessment’ section below*). Validation of self-reported socio-demographic and anthropometric measures has been reported previously [21].

### Objective physical activity assessment

#### Physical activity monitoring

PA was objectively assessed using the TracmorD tri-axial accelerometer (Philips Consumer Lifestyle, The Netherlands; http://www.directlife.philips.com) [22]. The device is small (3.2 × 3.2 × 0.5 cm), light (12.5 g), waterproof to a depth of 30 m, has a battery life of 3 weeks and an internal memory that can store data for up to 22 weeks. The accelerometer registers accelerations in the mediolateral (*x*-axis), longitudinal (*y*-axis) and anterioposterior (*z*-axis) axes [22] as the number of activity counts per minute.

In the present study, participants received the TracmorD accelerometer by post and activated it by creating an account online, installing an application on their computer and connecting the device to the computer using the USB-adapter provided. Upon activation, men could choose between 3 wearing positions (pocket, belt or necklace) and women between 4 wearing positions (pocket, belt, necklace or bra). Participants were instructed to wear the accelerometer every day during waking hours, except when taking a shower. Participants uploaded data by connecting their monitor to their computer. The data transferred were stored on a secured server.

#### Physical activity data processing

Data were recorded with a time sampling interval of 1 min (i.e. 1-min epochs). Sufficient PA data to be included in the analyses was defined as having at least 3 valid weekdays and 2 valid weekend days of accelerometer wear, since PA patterns may vary between week and weekend [23,24]. A day was considered valid if the participant had worn the TracmorD between 10–18 hours. Wear time was defined as 24 hours minus non-wear time. To define non-wear time, we adapted the recommendations of Choi et al. [25] to the TracmorD. Physical activity level (PAL) per minute and per day were estimated from activity counts [22]. Non-wear time was then defined by an interval of at least 90 consecutive minutes of PAL per minute values below 1.3889, allowing for 2-min interval of values above the threshold with the upstream or downstream 30-min window of consecutive values below the threshold for detection of artifactual movements. The R software [26] version 3.1.2 was used for all data handling.

### Physical activity variables

PA is presented in several ways: 1) daily PAL, 2) estimates of time spent in different PA intensities according to metabolic equivalent thresholds (METs), and 3) estimates of adherence to the latest WHO physical activity recommendation [9] and of adherence to the older 30 min · day^−1^ of MVPA recommendation [5] for comparison with previous studies.

PAL per day calculations are based upon that described by Bonomi *et al*. [22]. Mean PAL was calculated using all valid week and weekend days, as follows: mean = (mean for weekdays × 5 + mean for weekend days × 2) / 7.

Times spent in sedentary behavior, light PA, moderate PA, and vigorous PA were based on the application of thresholds for activity energy expenditure (AEE) corresponding to 1.5, 3 and 6 metabolic equivalents (METs). A MET represents the ratio of energy expended divided by resting energy expenditure and was estimated as 1 kcal · kg^−1^ · h^−1^ [27]. 1.5, 3 and 6 METs were therefore assumed to equal 1.5, 3, and 6 kcal · kg^−1^ · h^−1^ respectively or 0.025, 0.05 and 0.1 kcal · kg^−1^ · min^−1^. AEE per minute data were calculated as: (0.9 × PAL per minute– 1) × BMR / 1440, where PAL per minute was derived from accelerometer activity counts per minute, and BMR is the daily basal metabolic rate estimated using the Oxford equations developed by Henry, based on the participants’ sex, age and weight at baseline [28]. Sedentary time and light, moderate, and vigorous PA were then determined by summing minutes in a day where AEE per minute met the criterion for the appropriate intensity, and mean data were calculated using all valid week and weekend days as follows: mean = (mean for weekdays × 5 + mean for weekend days × 2) / 7.

Finally, to examine adherence to PA guidelines, moderate PA and vigorous PA duration data were also calculated for activity occurring in modified bouts of 10 min. A modified 10-min activity bout was defined as 10 or more consecutive minutes above the relevant threshold (3 or 6 METs), with allowance for interruptions of 1 or 2 min below threshold [29]. Adherence to the WHO PA recommendation was then examined by estimating the proportion of participants who accumulated at least 150 min · wk^−1^ of moderate PA or 75min · wk^−1^ of vigorous PA or an equivalent combination of MVPA, in modified 10-min bouts [9]. This can be more simply formulated as achieving at least 150 min · wk^−1^ of moderate-equivalent PA, in modified 10-min bouts, where moderate-equivalent PA is defined as moderate PA + (2 × vigorous PA). For comparison with studies that defined adherence to PA guidelines as accumulating 30 min · day^−1^ of MVPA in modified 10-min bouts, the proportion of participants achieving such amount of MVPA was also estimated. MVPA was calculated as moderate PA + vigorous PA.

### Statistical analyses

Data are presented by country for men and women separately. Categorical variables are given as percentages and continuous variables as adjusted mean ± standard deviation (SD) unless otherwise stated.

For all continuous variables, differences between men and women were examined using robust multiple linear regression models, based on computation of MM-type estimators [30], to account for the violation of the normality assumption. Differences in PA outcomes between countries were assessed with robust regression analyses stratified by sex. Models were adjusted for age, waist circumference (WC), season, accelerometer wear time and smoking. Significant associations between PA outcomes and country were further investigated using Tukey’s *post hoc* tests between adjusted means, to correct for multiple testing.

For men and women separately, differences in adherence to PA guidelines between countries were tested using binary logistic regression, with adjustments for age, WC, season, accelerometer wear time and smoking. Sensitivity analyses to compare dropouts with starters and compliant with less compliant individuals were carried out using robust t-tests (continuous variables) and chi-square tests (categorical variables).

All analyses were performed using the R software version 3.1.2 [26] and the significance level was set at *p* < 0.05.

## Results

### Compliance

From the original 1607 eligible participants recruited into the PoP study, 127 (8%) dropped out before the start of the intervention. These dropouts were more likely to be women (*p* = 0.014) and were significantly younger than the individuals who actually started the trial (*p* < 0.001). Neither group differed in BMI (data not shown). Of those who started the study, 43 (3%) did not wear the accelerometer—mainly due to incompatibilities between the accelerometer software and their personal computer at home or work. Thus, 1437 participants wore the monitors and had at least one day of accelerometer data available.

Of these 1437 individuals, 1092 (76%) had at least 3 valid weekdays and 2 valid weekend days of accelerometer wear in the 2 weeks baseline assessment period, (average number of valid days = 12 days, consisting of 9 weekdays and 3 weekend days). To maximize sample size, we extended the assessment period to 3 weeks for participants with insufficient valid days, allowing 195 (14%) additional individuals (average number of valid days = 9 days, 6 weekdays and 3 weekend days). These 195 additional individuals were more likely to be men (*p* = 0.012). They were younger (*p* < 0.003), had a slightly higher BMI (*p* = 0.05) and had lower PAL (*p* < 0.001) compared with individuals who had sufficient valid PA data in the 2 weeks assessment period (data not shown). The proportion of individuals requiring 3 weeks assessment period was highest in Greece and lowest in The Netherlands (*p* < 0.001, data not shown).

In total, 1287 individuals (age range: 18–79 years) were therefore included in the analyses (90% of participants who started wearing the accelerometer). Dutch participants were the most compliant with >97% having sufficient valid accelerometer data. In almost all countries, younger individuals (18–33 years) were less compliant than older participants (See S1 Table). Mean daily accelerometer wear time of the 1287 individuals included in the analyses was 14.4 h. Wear time was similar between countries and between men and women. However, participants aged 49+ years wore their accelerometer for more hours compared with younger individuals (data not shown). Compared with participants with sufficient PA data, individuals with some but insufficient data were younger (*p* < 0.001), but otherwise similar in BMI and were as likely to be men as women. However, the odds of having insufficient data were higher in Greece and lower in The Netherlands (*p* = 0.003). The characteristics of the analyzed sample are presented by sex and country in Table 1.

**Table 1:** Characteristics of the 1287 adults enrolled in the Food4Me Study, with sufficient accelerometer data^a^.

***A*.**	All	Germany	Greece	Ireland	Netherlands	Poland	Spain	UK
***MEN***	*n = 539*	*n = 80*	*n = 72*	*n = 76*	*n = 106*	*n = 57*	*n = 87*	*n = 61*
White (%)	95.9	98.8	97.2	98.7	94.3	100	95.4	86.9
Age (years)	41.3 (14.3)	46.3 (15.4)	38.1 (12.1)	40.4 (13.1)	46.3 (16.4)	35.1 (12.2)	42.6 (11.5)	39.5 (17.3)
Height (m)	1.79 (0.07)	1.82 (0.07)	1.75 (0.06)	1.78 (0.07)	1.82 (0.07)	1.77 (0.07)	1.76 (0.07)	1.77 (0.07)
Weight (kg)	82.1 (13.8)	82.2 (12.6)	82.6 (11.4)	85.8 (16)	80.1 (12.5)	84.3 (15.4)	83.2 (12.3)	80.8 (13.2)
BMI (*kg* · *m*^−2^)	25.7 (3.8)	24.8 (3.3)	26.7 (3.6)	26.8 (4.3)	24.1 (3.5)	26.7 (4.4)	26.7 (3.5)	25.4 (3.4)
*Overweight (%)*^b^	36.2	32.5	40.3	40.8	25.5	31.6	48.3	36.1
*Obese (%)*^c^	17.1	11.3	18.1	25	11.3	24.6	19.5	13.1
WC (cm)	91.3 (11.3)	90.4 (11)	95.4 (10.9)	94.4 (12.2)	87.5 (10.7)	93.4 (11.5)	93.5 (10.8)	90.3 (9.5)
Current smokers (%)	10.6	8.8	33.3	7.9	4.7	8.8	8	4.9
Ex-smokers (%)	31.5	33.8	26.4	28.9	38.7	17.5	41.4	24.6
Non-smokers (%)	57.9	57.5	40.3	63.2	56.6	73.7	50.6	70.5
***B*.**	All	Germany	Greece	Ireland	Netherlands	Poland	Spain	UK
***WOMEN***	*n = 748*	*n = 96*	*n = 103*	*n = 105*	*n = 108*	*n = 122*	*n = 94*	*n = 120*
White (%)	97.5	99	100	95.2	95.4	100	98.9	94.2
Age (years)	39.4 (13.8)	42.6 (14.9)	38.6 (12.2)	39.2 (13.5)	41.7 (17.3)	34.1 (13.6)	41.2 (10.7)	37.2 (14.1)
Height (m)	1.66 (0.06)	1.7 (0.07)	1.63 (0.06)	1.64 (0.06)	1.69 (0.07)	1.66 (0.07)	1.64 (0.06)	1.65 (0.06)
Weight (kg)	67.2 (14)	66.3 (11.1)	70.1 (15)	64.9 (14)	68 (12.1)	65.1 (13.4)	66.1 (12.2)	67.5 (14.3)
BMI (*kg* · *m*^−2^)	24.3 (4.9)	23.1 (3.6)	25.9 (5.7)	24 (4.6)	23.8 (3.9)	23.8 (4.5)	24.7 (4.3)	24.6 (4.9)
*Overweight (%)*^b^	25.1	20.8	33	21	28.7	21.3	30.9	21.7
*Obese (%)*^c^	15.5	8.3	26.2	14.3	12	13.1	17	17.5
WC (cm)	80.1 (12)	76.6 (8.9)	84.7 (15.6)	79.3 (10.5)	79.9 (10.5)	78.5 (12.6)	78.9 (10.4)	79.4 (11)
Current smokers (%)	11.9	5.2	35	3.8	4.6	8.2	23.4	5.8
Ex-smokers (%)	23	30.2	14.6	23.8	35.2	12.3	31.9	16.7
Non-smokers (%)	65.1	64.6	50.5	72.4	60.2	79.5	44.7	77.5

BMI, body mass index;

WC, waist circumference; n, number of individuals.

^a^Sufficient accelerometer data was defined as having at least 3 valid weekdays and 2 valid weekend days in the baseline assessment period.

^b^Overweight: BMI = 25.0–29.9 kg · m^−2^.

^c^Obese: BMI ≥ 30 kg · m^−2^. Data are presented as percentages for categorical variables and as adjusted means (standard deviations) for continuous variables. For each country, means of continuous variables are unadjusted (‘Age’) or adjusted for age (‘Height’, ‘Weight’, ‘BMI’, ‘WC’). For data in the column “all countries”, all means were in addition adjusted for country.

### Daily PAL

**Fig 1:**
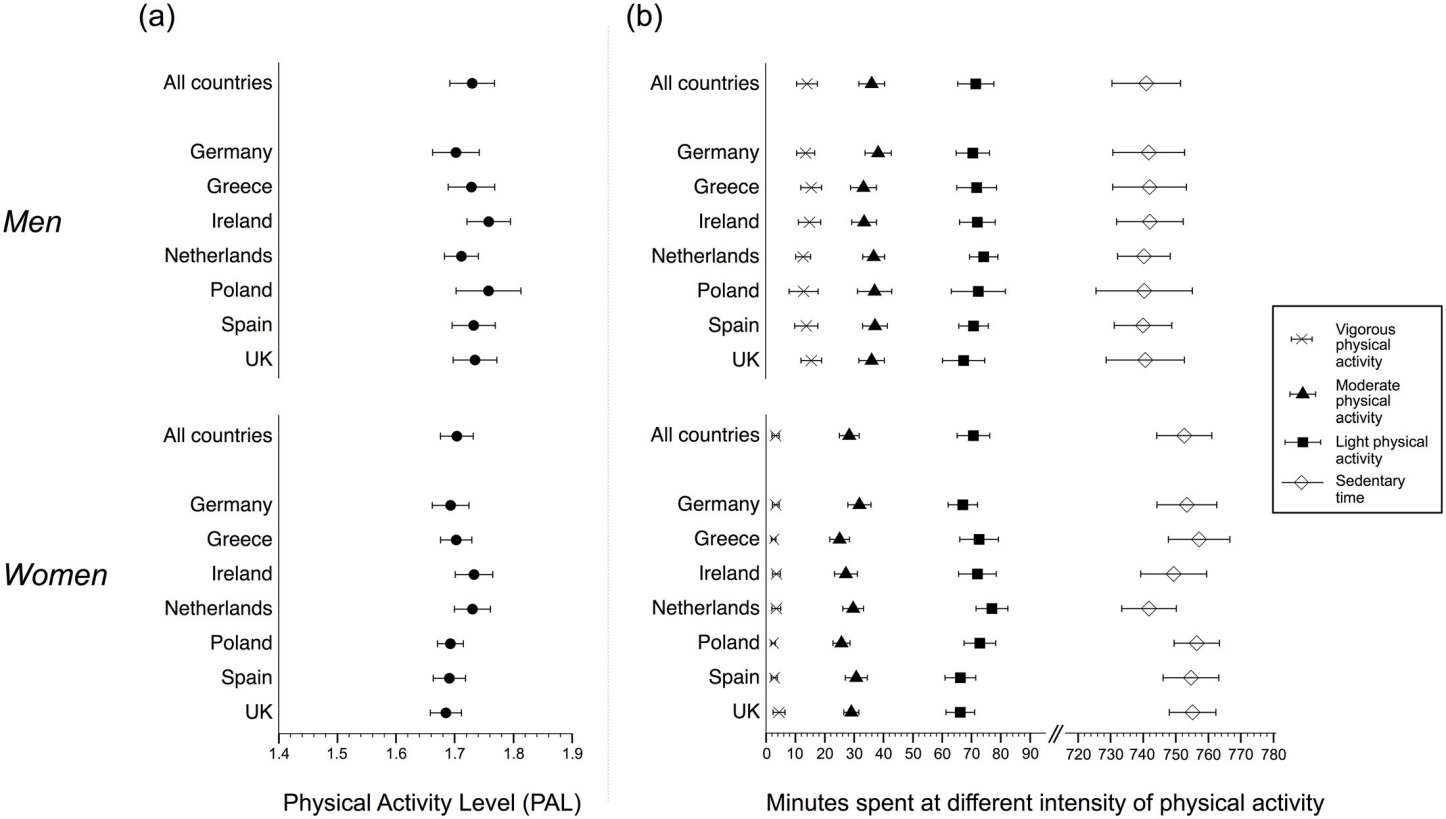
Physical activity level (a) and time spent at different intensity of physical activity (b) for the 1287 adults enrolled in the Food4Me Study, with sufficient accelerometer data^a^. ^a^Sufficient accelerometer data was defined as having at least 3 valid weekdays and 2 valid weekend days in the baseline assessment period. Means are adjusted for age, waist circumference, season, accelerometer wear time and smoking.

Men in the Food4Me cohort had significantly higher PAL than women (1.74 ± 0.19 vs. 1.70 ± 0.15, respectively, *p* < 0.001). For both sexes, there were no significant differences in PAL between countries (Fig 1a).

### Minutes of activity at different intensities

Women spent more time in sedentary behaviors (*p* < 0.0001) and less time in light PA (*p* = 0.003), moderate PA and vigorous PA (*p* < 0.0001) daily compared with men, achieving for example on average 4±9 min · d^−1^ vigorous PA compared with 11±25 min · d^−1^ for men (Fig 1b). For both sexes, less than half MVPA occurred in modified 10-min bouts (data not shown). Although all participants recorded some moderate PA, 3.2% of men and 6.1% of women had 0 min · d^−1^ of vigorous PA. Furthermore, 7.1% of men and 10.6% of women did not achieve any moderate PA in modified 10-min bouts, and 34.5% of men and 56.4% of women did not accumulate any vigorous PA in modified 10-min bouts (data not shown).

Greek and Polish women achieved significantly less vigorous PA than women in the UK (Fig 1b, *p* = 0.026 and *p* = 0.019, respectively). In addition, for women, there were some borderline significant inter-country differences in light PA (Fig 1b): light PA ranged from 66 min · d^−1^ in Spain and the UK to 77 min · d^−1^ in The Netherlands (NL) (Spain vs. NL, *p* = 0.065 and UK vs. NL, *p* = 0.053).

No significant differences between countries were observed for men (Fig 1b).

### Adherence to the PA guidelines

#### WHO PA recommendation

**Fig 2:**
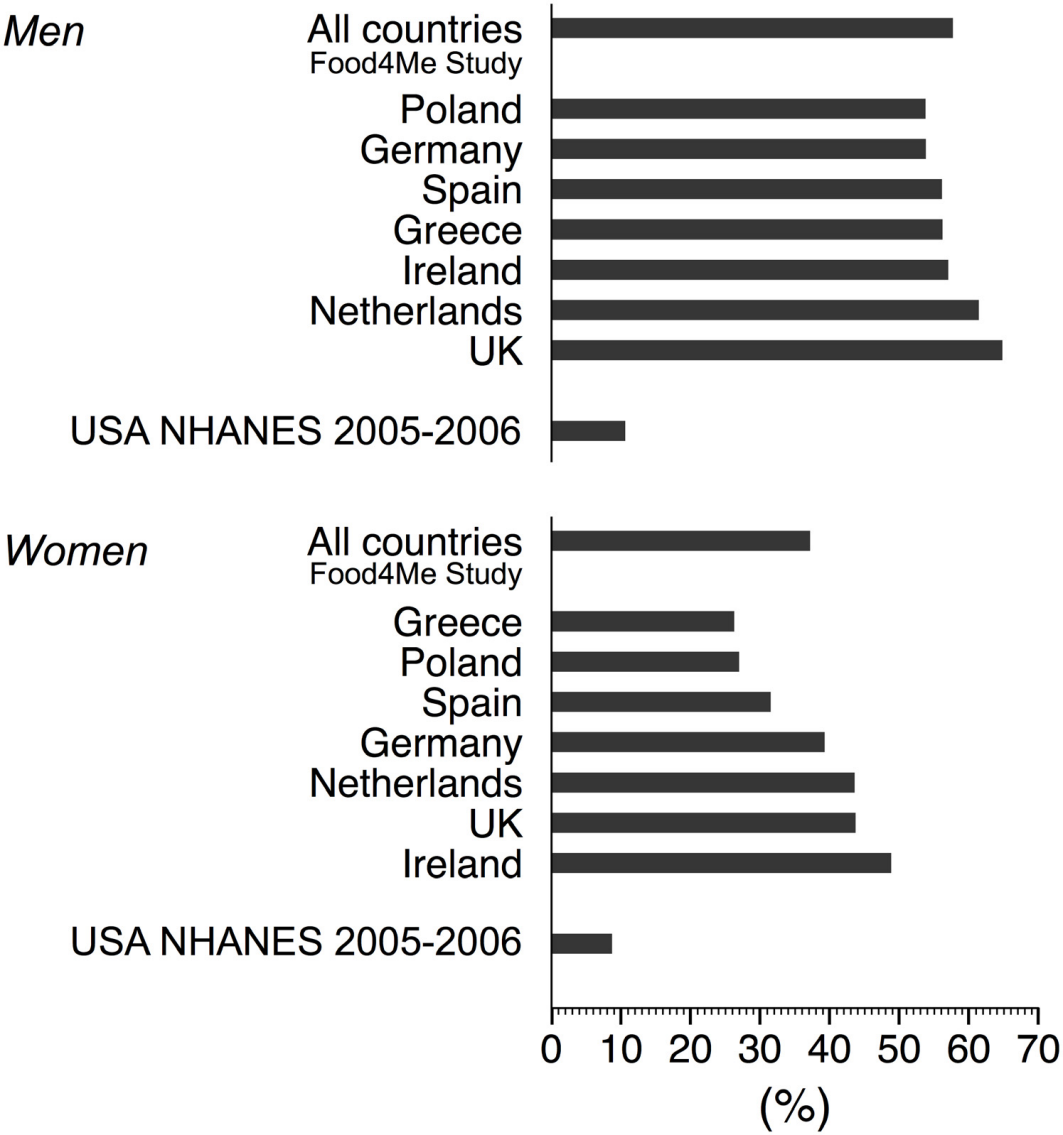
Proportion of subjects meeting the World Health Organization (WHO) Physical Activity (PA) recommendations by country. WHO PA recommendations are defined as accumulating a minimum of 150 min · wk^−1^ of moderate PA or 75 min · wk^−1^ of vigorous PA or an equivalent combination of moderate and vigorous PA, in modified 10-min bouts, i.e. bouts of 8–10 min. Mean prevalence estimates from the present study are adjusted for age, waist circumference, season, accelerometer wear time and smoking. Reference for USA NHANES 2005–2006: [31].

Among adults, 46.9% achieved the amount of PA recommended by the WHO [9], i.e. at least 150 min · wk^−1^ of moderate-equivalent PA in modified 10-min bouts (men: 57.7% vs. women: 37.2%; *p* < 0.0001, Fig 2). Women in Poland and Greece appeared less likely, while women in Ireland were more likely, to meet the WHO PA recommendations (borderline significance: *p* = 0.06 and *p* = 0.1, respectively; Fig 2 and S2 Table).

#### 30 min · d^−1^ PA recommendation

**Fig 3:**
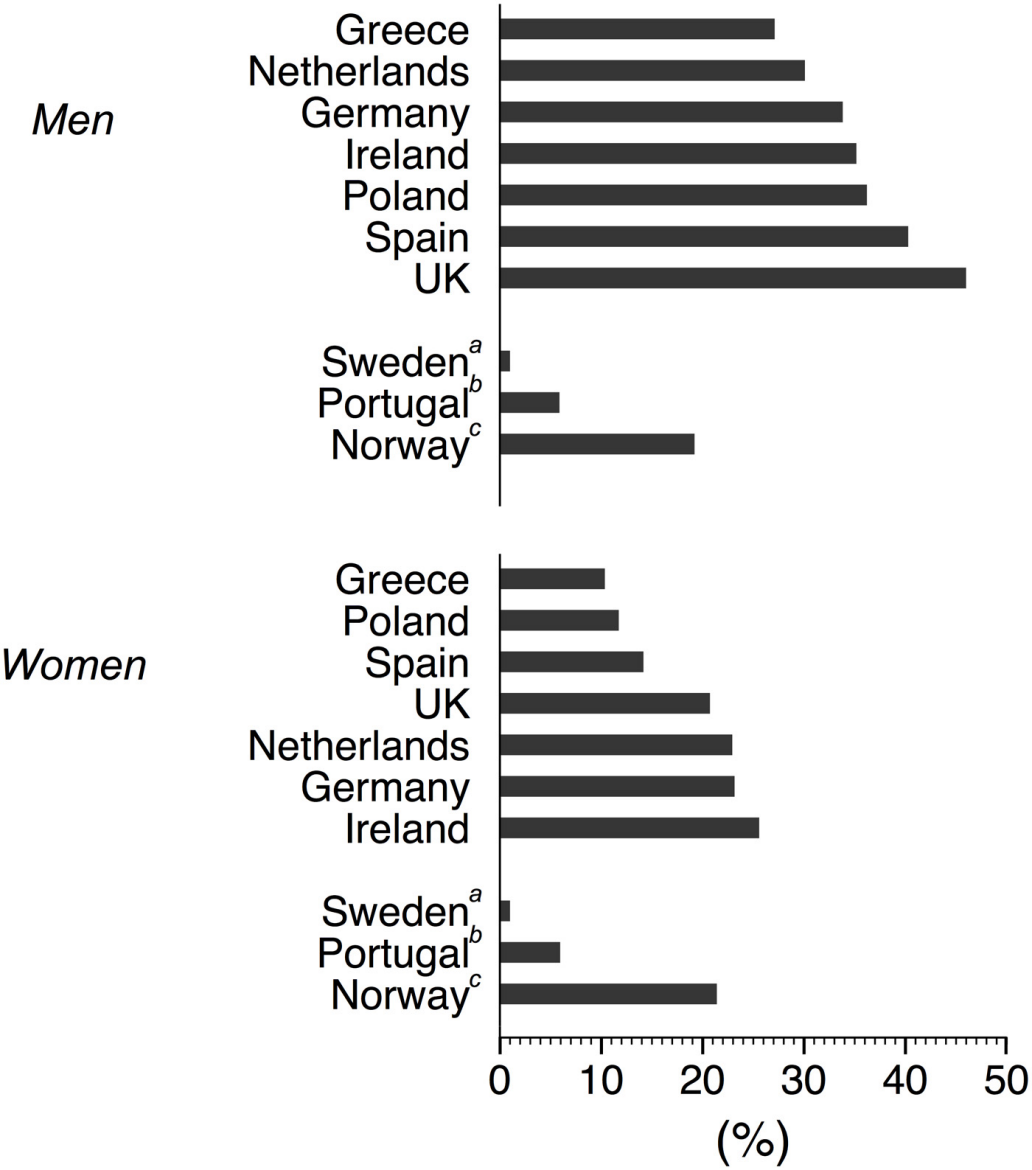
Proportion of subjects meeting the 30 min.d^-1^ Physical Activity (PA) recommendations by country. ^a^Swedish data: Hagstömer et al. [18]; ^b^Portuguese data: Baptista et al. [17]; ^c^Norwegian data: Hansen et al. [19]; Prevalence estimates from the present study are adjusted for age, waist circumference, season, accelerometer wear time and smoking.

When PA guidelines were defined as at least **3**0 min · **d**^−1^ of MVPA [5], prevalence estimates of meeting these guidelines were lower and ranged from 27.1% (Greece) to 46% (UK) for men and from 10.4% (Greece) to 25.6% (Ireland) for women. None of the inter-country differences reached statistical significance (Fig 3 and S3 Table).

All data relevant to the current analyses can be found in S4 Table.

## Discussion

The Food4Me Study demonstrated the feasibility of using Internet-collected and objectively measured physical activity data in large multi-country studies. In this trial we used the TracmorD accelerometer to compare PA among adults in 7 European countries.

The activity levels in our cohort are in line with what is expected for the average EU population. In 2010, Speakman and Westerterp described PAL data for more than 500 Dutch men and women above 18 years, using the gold standard doubly labeled water technique [32]. They showed an average PAL of 1.83 and 1.71 for men and women aged 18–69, respectively. Earlier, Black et al. had published similar PAL values after summarizing most doubly labeled water data available worldwide at the time (PAL of 1.79 and 1.69 for men and women aged 18–64, respectively) [33]. These data are comparable with our results. We found sex differences in PAL as well as in time spent in different activities. Men spent more time in MVPA and less time in sedentary behaviors than women, which is in line with the literature [4].

Furthermore, for women, we observed some between-country differences especially in vigorous PA. Consequently, there was a trend for women in Poland and Greece to be less likely to meet the WHO PA guidelines, compared with Irish women. Women in Greece and Poland reported doing less sport and being less active at work, compared with women in Ireland, which may explain in part these differences. Notably, 23% of female participants in Greece were housewives compared with 3–15% in the other countries data not shown).

The prevalence estimates of meeting the WHO guidelines reported for our sample are much higher than the 10.6% and 8.7% reported for US men and women respectively by Tucker et al. [31] using NHANES 2005–2006 data (Fig 2). European adults may be more active than US adults [34]. For example, it has been shown that people in The Netherlands and Germany, but also UK and Ireland walk or cycle to work substantially more than in the USA [4]. However, part of the large differences observed may be because our sample is composed of self-selected individuals, interested in personalized nutrition and likely to be more health conscious and therefore more active, whereas the NHANES cohort is more representative of the general US population and includes a wider variety of individuals in terms of ethnicities, and socioeconomic status. In addition, prevalence of overweight and obesity, which is associated with lower PA, is higher in the USA than in most European countries.

Recent studies of proportions of European individuals meeting PA recommendations based on objectively measured PA are scarce. Estimates of adherence to PA guidelines based on accelerometers have been reported for Sweden [18], Norway [19], and Portugal [17]. However, meeting PA guidelines in these studies was defined as achieving 30 min of MVPA daily in 10-min bouts. Thus, for comparison purposes, we also calculated our estimates based on this definition because results have been shown to vary according to how meeting the guidelines is defined [31]. We observed that substantially more men in the Food4Me Study achieved **3**0 min · **d**^−1^ of MVPA, but our results were comparable with the Portuguese [17] and Norwegian [19] studies for women (Fig 3). The Swedish study was conducted in 2001, which may partly explain the lower prevalence estimates of adherence [18]. Public awareness of increasing PA may have increased since then.

Running a large-scale study remotely is challenging, especially when there is an absence of face-to-face contact with the participants. Yet, compliance was good (90% of participants completed the measurement). The number of issues was acceptable (<15% of participants reported issues on average across all seven centers, range 5–20%): there were very few logistical problems in the distribution of the monitors directly to participants’ homes across seven European countries (5 monitors lost during shipment across all centers); accelerometers were tested before shipment and <1% were returned and replaced due to malfunction (battery problem or defect USB adapter); the widest majority of participants could activate their device without support beyond the instructions provided (90%)–there were some cases (<10%) where the invitation email to activate the monitor had to be resent, or where the antivirus program on a participant’s personal computer would not allow him/her to install the application required, but issues were rapidly solved. Researchers’ quick responsiveness to participants’ email enquiries, and their routine checks that monitors were activated and recording data properly, were key elements in the success of implementing this remote collection of accelerometer data via the Internet among participants recruited nation-wide in seven European countries.

### Strengths and limitations

Use of objective measurement monitors, remotely uploaded data and a high compliance rate were strengths of this study. Overall, our study shows that it is feasible to use accelerometers to collect PA data using the Internet in a large group of individuals, in multiple countries.

A limitation of our study is that the participants who joined the study were self-selected resulting in an element of selection bias as in many lifestyle intervention studies, including those which are web-based [35]. In the French NutriNet-Santé study, the recruited sample included proportionally 3 times as many individuals of relatively high socio-economic status as compared with national estimates [36]. The Food4Me PoP study required that participants be able to use a computer and Internet. Furthermore, by design, we recruited “health-seeking” individuals with an interest in personalized nutrition. These individuals are likely more active than the general population, which makes our results less generalizable compared with a European-wide, population representative, survey. Being part of the study and doing the measurements (e.g. self-weighing, wearing the accelerometer) could have influenced participants’ physical activity pattern. However, this is a common reaction to behavioral interventions.

In addition, although accelerometry is an objective measure of PA, it does not capture well muscle-strengthening activities (such as lifting weights), which are, next to aerobic activities, another important dimension of PA recommendations. Accelerometers can also underestimate activities such as cycling. This may affect our results especially when making comparisons between countries, as people cycle more in The Netherlands, for example [4]. Whilst the TracmorD was able to measure body movement in three axes, it does not discriminate between walking uphill or on a flat surface. The device is waterproof however, and was used during swimming, but this activity may also be underestimated in terms of activity intensity. Furthermore, as pointed out by Troiano and colleagues in a recent review, evaluation of PA guidelines adherence based on accelerometer outcomes may be inappropriate because current guidelines were developed based on self-reports and not on accelerometer data [37]. Although this matters less when comparing between countries, PA guidelines based on accelerometer-derived measures are still urgently needed. A global repository of the rapidly growing pool of accelerometry data may be very helpful in this respect [16]. Self-reported PA and accelerometer-measured PA are not interchangeable but, in general, accelerometers provide more reliable data than self-reports [13,14]. The TracmorD used in this study has been validated against doubly labeled water and compared with other accelerometers such as those used in the studies mentioned above, and several publications show that it is a reliable and accurate monitor [22,38–40]. Accelerometers are becoming more pervasive as a tool for both surveys and for interventions aiming to promote public health and may be useful in motivating individuals to increase their PA.

## Conclusions

We observed some inter-country differences in PA in participants in the Food4Me Study. The majority of men but not women met the WHO recommendations for PA. For all countries, fewer individuals achieved 30 minutes of MVPA daily. Thus, a large part of participants, especially women, would benefit from greater levels of PA. Finally, we showed that using accelerometers is feasible and the resultant data can be collected remotely via the Internet in large multi-country surveys and interventions.

## Supporting Information

S1 Table Proportions of participants with sufficient valid accelerometer data. (PDF)

S2 Table Results from the binary logistic regression model examining the association between meeting the World Health Organization (WHO) physical activity recommendation and predictor variables in 539 men and 748 women. (PDF)

S3 Table Results from the binary logistic regression model examining the association between meeting the 30 min.d-1 physical activity recommendation and predictor variables in 539 men and 748 women. (PDF)

S4 Table Data relevant to the current analyses. (CSV)
