# Supplementary material for: Objectively Measured Physical Activity in European Adults: Cross-Sectional Findings from the Food4Me Study
Source: PLoS One. 2016 Mar 21;11(3):e0150902. doi: 10.1371/journal.pone.0150902 (PMC4801355; doi:10.1371/journal.pone.0150902)
Supplement: S1 Table — (PDF) [file pone.0150902.s001.pdf]

S1 Table. Relative proportions (%) of participants with at least 3 valid[a] weekdays and 2 valid weekend days of accelerometer wear from the total number of participants with any accelerometer data (1437 individuals).

	MEN								WOMEN							
	**All**	**Germany**	**Greece**	**Ireland**	**NL**	**Poland**	**Spain**	**UK**	**All**	**Germany**	**Greece**	**Ireland**	**NL**	**Poland**	**Spain**	**UK**
Total	89.8	87.9	87.8	90.5	96.4	96.6	84.5	85.9	89.4	88.9	84.4	84.7	98.2	87.8	93.1	90.2
Age (years)																
18-33	84.1	84.6	81.1	89.7	88.9	96.6	73.1	75.8	86.3	82.1	84	75.9	97.4	88.2	93.3	87.1
34-48	91.5	85	93.3	87.1	96.9	100	89.1	90	90	90.3	82.9	86.1	100	91.2	93	89.7
49+	94.3	91.1	93.3	95.8	100	90	87.1	100	93.2	94.7	87.1	97.1	97.8	82.8	92.9	96.9
BMI ($kg.m^{-2}$)																
<25.0	89.7	91.8	83.3	86.7	94.4	96.2	84.8	86.1	89.2	89.5	80.8	84	100	87.9	92.5	90.1
25.0-29.9	87.1	78.8	87.9	88.6	100	94.7	85.7	78.6	89	90.9	87.2	80.8	100	76.5	100	89.7
30+	92.9	100	100	100	100	100	73.9	88.9	86.7	80	87.1	94.1	86.7	80	84.2	91.3

BMI, body mass index; NL, The Netherlands; UK, United Kingdom

[a]A valid day was defined as having 10 to 18 hours of accelerometer wear
