# Supplementary material for: Objectively Measured Physical Activity in European Adults: Cross-Sectional Findings from the Food4Me Study
Source: PLoS One. 2016 Mar 21;11(3):e0150902. doi: 10.1371/journal.pone.0150902 (PMC4801355; doi:10.1371/journal.pone.0150902)
Supplement: S2 Table — (PDF) [file pone.0150902.s002.pdf]

S2 Table. Results from the binary logistic regression model examining the association between meeting the World Health Organization (WHO) physical activity recommendations and predictor variables in 539 men and 748 women.

	MEN	WOMEN
	**Odds ratio (95%CI)**	**Odds ratio (95%CI)**
Intercept	-	-
Country: Netherlands	Ref	Ref
Country: Germany	0.73 (0.39, 1.39)	0.84 (0.45, 1.54)
Country: Greece	0.81 (0.4, 1.64)	0.46 (0.23, 0.89)
Country: Ireland	0.83 (0.42, 1.66)	1.24 (0.64, 2.37)
Country: Poland	0.73 (0.33, 1.61)	0.48 (0.26, 0.88)
Country: Spain	0.80 (0.43, 1.5)	0.60 (0.32, 1.11)
Country: UK	1.16 (0.56, 2.45)	1.01 (0.56, 1.8)
Age	1.00 (0.99, 1.02)	0.98 (0.96, 0.99)
Waist circumference	0.95 (0.94, 0.97)	0.94 (0.92, 0.96)
Season: winter	Ref	Ref
Season: autumn	0.56 (0.2, 1.5)	1.57 (0.77, 3.27)
Season: spring	1.47 (0.93, 2.32)	1.42 (0.98, 2.07)
Season: summer	1.05 (0.47, 2.36)	1.44 (0.71, 2.89)
Accelerometer wear time	0.80 (0.67, 0.96)	1.08 (0.92, 1.28)
Smoking: non-smokers	Ref	Ref
Smoking: ex-smokers	0.84 (0.55, 1.28)	1.21 (0.8, 1.83)
Smoking: current smokers	0.24 (0.12, 0.47)	0.59 (0.32, 1.04)

CI, confidence interval

MEN: Model $\chi^2(14)=81.73$, $p<0.0001$; WOMEN: Model $\chi^2(14)=131.91$, $p<0.0001$
