# Supplementary material for: Objectively Measured Physical Activity in European Adults: Cross-Sectional Findings from the Food4Me Study
Source: PLoS One. 2016 Mar 21;11(3):e0150902. doi: 10.1371/journal.pone.0150902 (PMC4801355; doi:10.1371/journal.pone.0150902)
Supplement: S3 Table — (PDF) [file pone.0150902.s003.pdf]

S3 Table. Results from the binary logistic regression model examining the association between meeting the 30 min.d^{-1} physical activity recommendation and predictor variables in 539 men and 748 women.

	**MEN**	**WOMEN**
	**Odds ratio (95%CI)**	**Odds ratio (95%CI)**
Intercept	-	-
Country: Netherlands	Ref	Ref
Country: Germany	1.19 (0.63, 2.25)	1.01 (0.51, 2)
Country: Greece	0.86 (0.41, 1.78)	0.39 (0.16, 0.89)
Country: Ireland	1.26 (0.63, 2.52)	1.16 (0.56, 2.37)
Country: Poland	1.32 (0.59, 2.9)	0.45 (0.22, 0.9)
Country: Spain	1.57 (0.84, 2.93)	0.55 (0.25, 1.18)
Country: UK	1.98 (0.99, 4)	0.88 (0.45, 1.7)
Age	1.01 (0.99, 1.03)	0.99 (0.97, 1)
Waist circumference	0.95 (0.93, 0.97)	0.94 (0.92, 0.96)
Season: winter	Ref	Ref
Season: autumn	0.40 (0.12, 1.2)	1.78 (0.82, 3.84)
Season: spring	1.48 (0.93, 2.37)	1.77 (1.14, 2.8)
Season: summer	1.37 (0.6, 3.1)	1.86 (0.81, 4.12)
Accelerometer wear time	0.85 (0.71, 1.02)	1.15 (0.95, 1.4)
Smoking: non-smokers	Ref	Ref
Smoking: ex-smokers	0.81 (0.53, 1.24)	1.01 (0.62, 1.62)
Smoking: current smokers	0.40 (0.18, 0.84)	0.37 (0.15, 0.82)

CI, confidence interval

MEN: Model $\chi^2(14)=64.66$, $p<0.0001$; WOMEN: Model $\chi^2(14)=102.20$, $p<0.0001$
